# Removal of intramural trapped intrauterine device by cystoscopic incision of bladder wall

**DOI:** 10.1590/S1677-5538.IBJU.2018.0056

**Published:** 2019-04-01

**Authors:** Abbas Basiri, Behnam Shakiba, Niloufar Rostaminejad

**Affiliations:** 1 Urology and Nephrology Research Center, Shahid Labbafinejad Hospital, Shahid Beheshti University of Medical Sciences, Tehran, Iran

## Abstract

A healthy 37 - year - old woman referred to our clinic with one - year history of recurrent urinary tract infection, dysuria and frequency. Her past medical history informed us that an IUD (Copper TCu380A) had been inserted 11 years ago. Eleven months after the IUD insertion she had become pregnant, unexpectedly. At that time, she had undergone gynecological examination and abdominal ultrasound study. However, the IUD had not been found, and the gynecologist had made the diagnosis of spontaneous fall out of the IUD. She had experienced normal pregnancy and caesarian section with no complications.

On physical examination, pelvic examination was normal and no other abnormalities were noted. Urinalysis revealed microhematuria and pyuria. Urine culture was positive for Escherichia coli. Ultrasound study revealed a calculus of about 10 mm in the bladder with a hyperdense lesion. A plain abdominal radiograph was requested which showed a metallic foreign body in the pelvis. We failed to remove the IUD by cystoscopic forceps because it had strongly invaded into the uterine and bladder wall. Despite previous papers suggesting open or laparoscopic surgeries in this situation (1, 2), we performed a modified cystoscopic extraction technique. We made a superficial cut in the bladder mucosa and muscle with J - hook monopolar electrocautery and extracted it completely with gentle traction.

This technique can decrease the indication of open or laparoscopic surgery for extraction of intravesical IUDs. In the other side of the coin, this technique may increase the risk of uterovesical fistula. Therefore, the depth of incision is important and the surgeon should cut the bladder wall superficially with caution. Although present study is a case report which is normally classified as with low level of evidence, it seems that our modified cystoscopic extraction technique is a safe and useful method for extraction of partially intravesical IUDs.

## ARTICLE INFO

**Available at: http://www.intbrazjurol.com.br/video-section/20180056_Basiri_et_al**

**Int Braz J Urol. 2019; 45 (video #6): 408-9**
